# Functional cardiac consequences of β-adrenergic stress-induced injury in a model of Duchenne muscular dystrophy

**DOI:** 10.1242/dmm.050852

**Published:** 2024-10-09

**Authors:** Conner C. Earl, Areli J. Javier, Alyssa M. Richards, Larry W. Markham, Craig J. Goergen, Steven S. Welc

**Affiliations:** ^1^ Weldon School of Biomedical Engineering, Purdue University, West Lafayette, IN 47907, USA; ^2^ Department of Medicine, Indiana University School of Medicine, IN 46202, USA; ^3^ Musculoskeletal Health Sciences Program, Indiana University School of Medicine, Indianapolis, IN 46202, USA; ^4^ Division of Pediatric Cardiology, Riley Children's Hospital at Indiana University Health, Indiana University School of Medicine, Indianapolis, IN 46202, USA; ^5^ Department of Anatomy, Cell Biology and Physiology, Indiana University School of Medicine, Indianapolis, IN 46202, USA; ^6^ Indiana Center for Musculoskeletal Health, Indianapolis, IN 46202, USA

**Keywords:** Duchenne muscular dystrophy, *mdx*, Isoproterenol, 4DUS, Cardiac strain, Mouse model

## Abstract

Cardiomyopathy is the leading cause of death in Duchenne muscular dystrophy (DMD); however, in the *mdx* mouse model of DMD, the cardiac phenotype differs from that seen in DMD-associated cardiomyopathy. Although some have used pharmacologic stress to stimulate injury and enhance cardiac pathology in the *mdx* model, many methods lead to high mortality with variable cardiac outcomes, and do not recapitulate the structural and functional cardiac changes seen in human disease. Here, we describe a simple and effective method to enhance the cardiac phenotype model in *mdx* mice using advanced 2D and 4D high-frequency ultrasound to monitor cardiac dysfunction progression *in vivo*. *mdx* and wild-type mice received daily low-dose (2 mg/kg/day) isoproterenol injections for 10 days. Histopathological assessment showed that isoproterenol treatment increased myocyte injury, elevated serum cardiac troponin I levels and enhanced fibrosis in *mdx* mice. Ultrasound revealed reduced ventricular function, decreased wall thickness, increased volumes and diminished cardiac reserve in *mdx* compared to wild-type mice. Our findings highlight the utility of challenging *mdx* mice with low-dose isoproterenol as a valuable model for exploring therapies targeting DMD-associated cardiac pathologies.

## Teaser

**Summary:** This work introduces an improved method to model heart failure in mouse models of Duchenne muscular dystrophy and comprehensively describes underlying cellular and physiologic mechanisms using advanced imaging techniques.

## INTRODUCTION

Duchenne muscular dystrophy (DMD) is a progressive X-linked skeletal and cardiac myopathy affecting 1 in 5000 live male births (Romitti et al., 2015). It is caused by mutation of the dystrophin gene, resulting in the loss of functional protein (Hoffman et al., 1987). Dystrophin is a large protein found at the inner surface of the sarcolemma linking the actin cytoskeleton to the cytosolic surface of muscle cell membranes providing structural support. The loss of dystrophin leads to striated muscle membrane weakness with increased susceptibility to damage caused by mechanical stress (Petrof et al., 1993). Myocyte damage can lead to cell death, chronic inflammation and fibrotic replacement of contractile tissue, further contributing to the destruction of muscles and magnitude of the disease (Tidball et al., 2018). Clinically, DMD patients present with skeletal muscle weakness and wasting, impaired motor function, loss of ambulation, respiratory insufficiency and, eventually, pronounced cardiac disease (Birnkrant et al., 2018a,b; McNally et al., 2015). Progress in treating respiratory insufficiency and complications related to the deterioration of skeletal muscles has extended lifespan, leading to cardiomyopathy being the primary life-limiting factor affecting DMD patients (Eagle et al., 2007; Kamdar and Garry, 2016; Nigro et al., 1990).

The *mdx* mouse, which carries a spontaneous mutation that results in the absence of dystrophin, is the most widely used pre-clinical animal model of DMD (Bulfield et al., 1984; McGreevy et al., 2015). Muscles from DMD patients and *mdx* mice feature asynchronous muscle damage, chronic inflammation, fibro-fatty degeneration and diminished function (McGreevy et al., 2015). However, these clinical symptoms are less severe in *mdx* mice than in DMD patients, exemplified by only a minor reduction in lifespan (Chamberlain et al., 2007). In particular, cardiac disease occurs especially late in the lifespan of *mdx* mice relative to human disease. Modest changes in left-ventricular function frequently are not detected until 9-12 months of age in *mdx* mice, whereas nearly 60% of patients have some degree of left-ventricular functional impairment by the age of 18 (James et al., 2020; Quinlan et al., 2004; Spurney et al., 2008; Stuckey et al., 2012). A limitation of the *mdx* mouse is that even with advanced age they do not develop dilated cardiomyopathy as seen in DMD patients (Yucel et al., 2018). To advance the disease state, the genetic background of mice carrying the *mdx* mutation has been altered. The DBA/2J (D2)-*mdx* murine model demonstrates worsened skeletal and cardiac muscle pathologies (Coley et al., 2016; van Putten et al., 2019). However, the DBA/2J mouse strain is susceptible to spontaneous cardiac calcinosis pathologies (Eaton et al., 1978) and its suitability as a model for DMD is debated (Hakim et al., 2017; Hart et al., 2022). Alternatively, double-knockout mouse models have been generated that combine the dystrophin mutation of *mdx* mice with an additional gene mutation, such as the *mdx*-*Utrn* double-knockout mouse model, which also exhibits worsened cardiac pathologies (Grady et al., 1997). Additional targeted mutations, however, can lead to a different molecular basis of disease from that which occurs in DMD patients (Yucel et al., 2018). With cardiomyopathy becoming a primary cause of morbidity and mortality in DMD patients, representative pre-clinical models are critically important for testing novel therapeutics and pathological mechanisms related to cardiac disease (Lechner et al., 2023).

Stressors that increase cardiac workload can induce cardiac myocyte injury and a more severe pathology in *mdx* mice. The β-adrenergic agonist isoproterenol has become a commonly used pharmacological stimulus of sarcolemmal injury in dystrophic cardiac myocytes (Danialou et al., 2001; Meyers et al., 2019; Spurney et al., 2011; Yue et al., 2004). Isoproterenol induces a sudden increase in heart rate (HR) and contractility stimulating mechanical stress resulting in prominent injury and eventual fibrotic replacement of myocardial tissue (Danialou et al., 2001; Meyers et al., 2019; Spurney et al., 2011). This pharmacological approach may be advantageous to other means of elevating cardiac work, such as exercise, owing to the low capacity of *mdx* mice for physical activity, and may better recapitulate human DMD without introducing additional confounding genetic variables (Betts et al., 2015). However, isoproterenol dosing concentrations, routes of administration and reported cardiac outcomes are highly variable and, in many cases, lead to early mortality (Elsherif et al., 2008; Lu and Hoey, 2000; Sarma et al., 2010; Spurney et al., 2011; Sullivan et al., 2021; Yue et al., 2004).

**Fig. 1.**
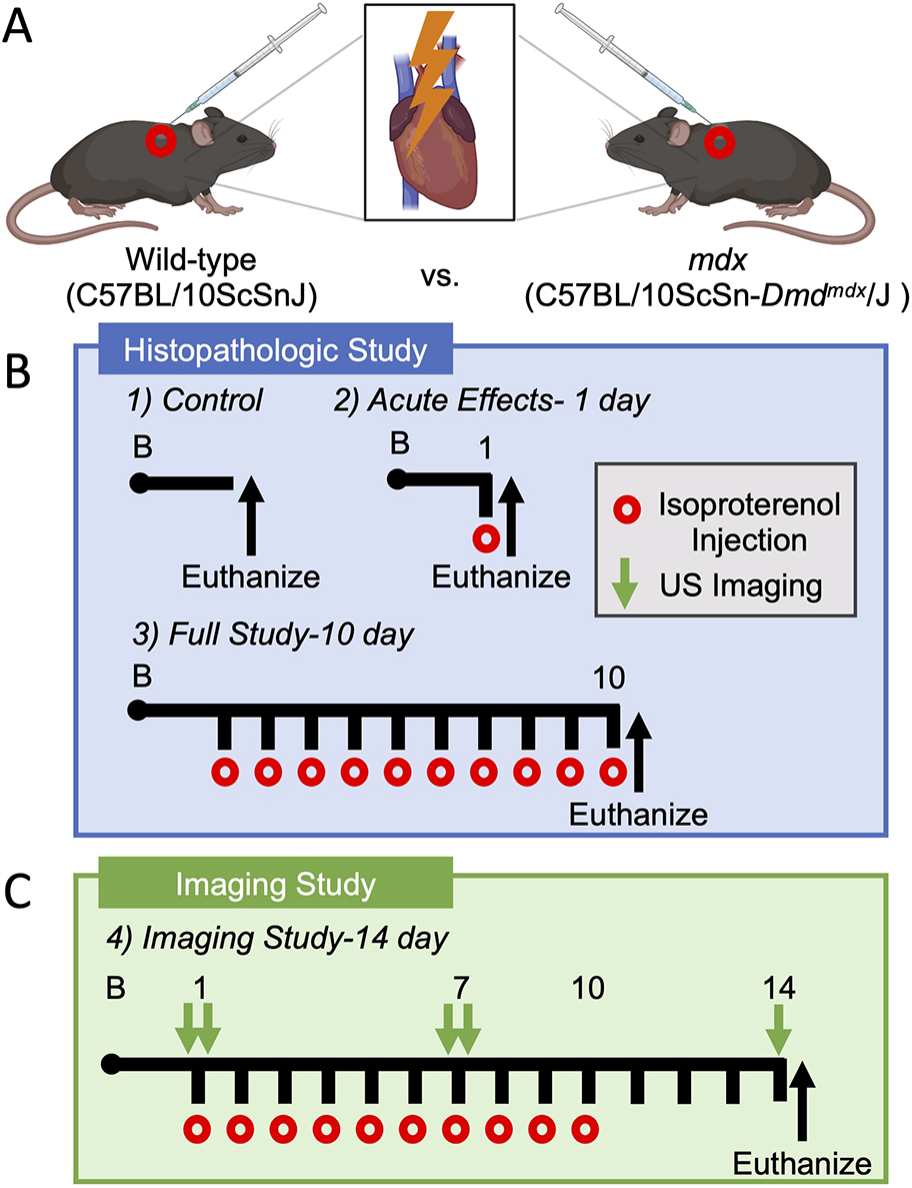
**Study overview.** (A) Schematic representing subcutaneous injection of 2 mg/kg/day isoproterenol to induce β-adrenergic cardiac stimulation and injury. (B) Histopathological study timeline. (C) Imaging study timeline. US, ultrasound.

In this study, we performed a comprehensive evaluation of cardiac injury, remodeling, function and their dynamics to isoproterenol challenge in wild-type and *mdx* mice. We tested for these changes at 10-12 weeks of age, before the clinical onset of cardiac disease in *mdx* mice, focusing on adaptive changes to isoproterenol-induced injury before the development of verified pathologies (Quinlan et al., 2004; Spurney et al., 2008; Stuckey et al., 2012). We utilized two-dimensional (2D) and four-dimensional (4D) high-frequency ultrasound imaging to test relative changes in left ventricle morphology and function with isoproterenol challenge over time (Fig. 1). We also report, for the first time, changes in β-adrenergic responsiveness by using ultrasound to measure acute changes in function before and after isoproterenol injection at baseline and after 7 days of isoproterenol challenge. Additional experiments assayed for changes in the molecular stress response, myocardial damage and fibrosis with acute and chronic isoproterenol treatment. Overall, our findings support that dystrophin-deficient cardiac myocytes are acutely susceptible to isoproterenol injury, resulting in distinct pathological and functional outcomes consistent with features of cardiac disease in DMD patients.

## RESULTS

### Dystrophin-deficient cardiac myocytes are susceptible to acute sarcolemmal injury with isoproterenol treatment

**Fig. 2.**
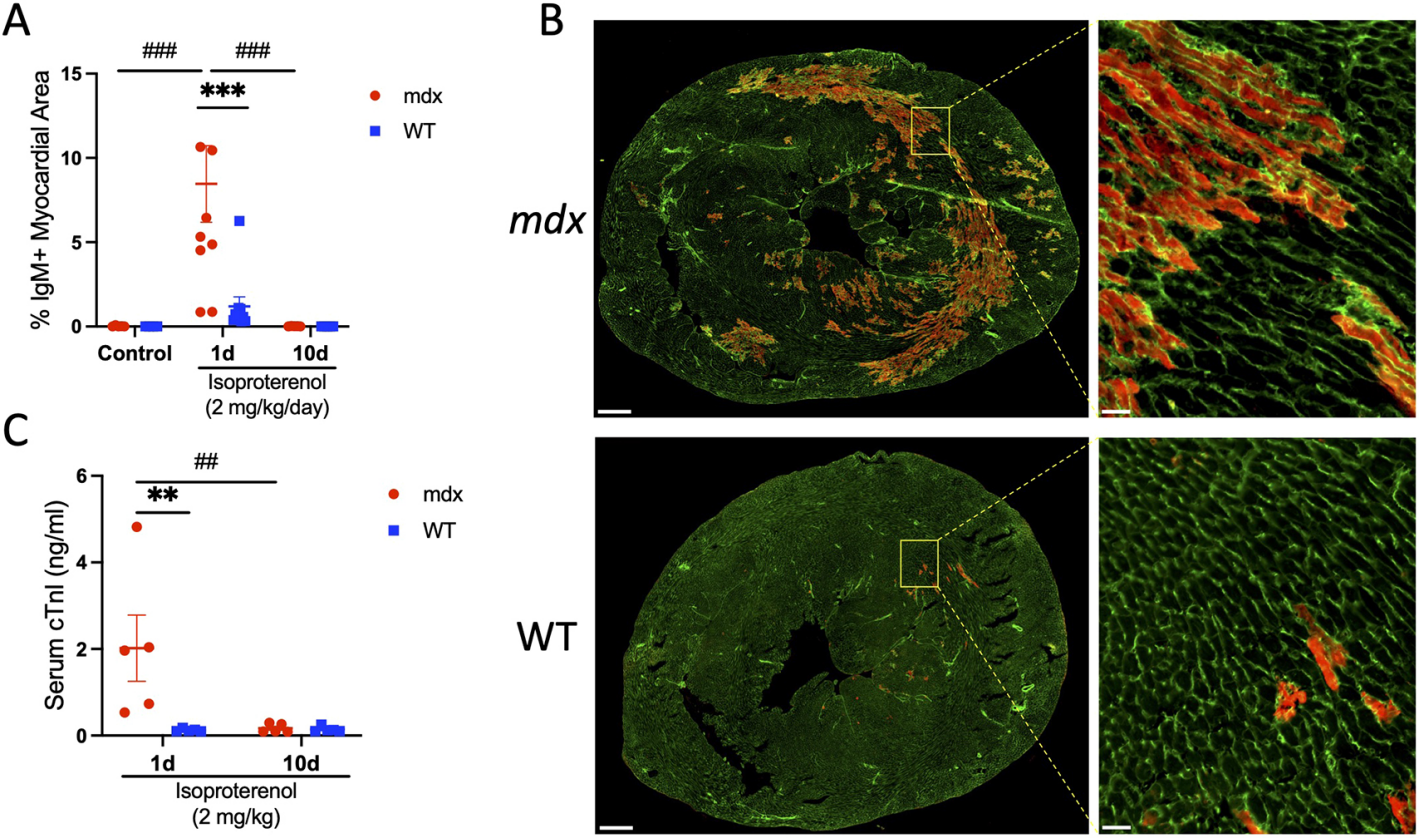
**Isoproterenol promotes sarcolemmal injury in dystrophin-deficient cardiac myocytes.** The acute and chronic effects of isoproterenol (2 mg/kg/day) were assessed in *mdx* and wild-type (WT) mice treated for 1 (*n*=10 per group) or 10 (*n*=5 per group) days. Control mice were injected with an equal volume of saline for 10 days (*n*=5 *mdx* and *n*=4 WT). (A,B) To assess sarcolemmal damage, mid-chamber coronal sections of ventricles were immunolabeled with anti-IgM (red) and counterstained with wheat germ agglutinin (WGA; green) to visualize tissue morphology. (A) Cardiac injury was detected transiently in *mdx* mice after isoproterenol challenge, with IgM^+^ cardiac myocytes occupying ∼8.5% of the myocardial area after a single isoproterenol treatment. Cardiac myocyte injury was scarcely detected in *mdx* mice after chronic isoproterenol administration or in WT mice across all conditions. (B) Representative *mdx* (top row) and WT (bottom row) whole-ventricle cross-section and high-magnification images show prominent areas of injury (red) after acute isoproterenol treatment in *mdx* mice. Scale bars: 500 µm. Insets: high-magnification images confirm that the IgM^+^ signal is localized to cardiac myocytes in *mdx* mice. Scale bars: 50 µm. (C) Serum cardiac troponin I (cTnI) levels were measured by ELISA as an independent assay of cardiac injury. Serum cTnI levels were also transiently elevated in *mdx* mice after acute isoproterenol treatment (*n*=5 per group). Data are presented as mean±s.e.m. All *P-*values are based on two-way ANOVA with Tukey's multiple comparison test. **P<0.01 and ***P<0.001 versus WT within a treatment condition. ^##^*P*<0.01 and ^###^*P*<0.001 between treatment groups within a genotype. d, day.

The loss of dystrophin is associated with increased occurrences of cardiac myocyte damage, necrosis and fibrotic replacement of myocardial tissue. We examined cardiac myocyte damage in control *mdx* mice at 10-12 weeks of age (Fig. 1A,B). Immunofluorescence analysis of mid-chamber myocardial cross-sections showed trace detection of dystrophic cardiac myocytes with IgM inclusion, indicating that sarcolemmal injury in *mdx* mice does not differ from that in wild-type mice at this age (Fig. 2A). Next, wild-type and *mdx* mice were injected with a single dose of isoproterenol (Fig. 1B). Isoproterenol increases mechanical stress and is a well-accepted model of sarcolemmal injury in dystrophic mice (Danialou et al., 2001). Within 24 h of acute isoproterenol (2 mg/kg) treatment, ∼8.5% of the myocardium was occupied by damaged IgM^+^ cardiac myocytes in *mdx* mice (Fig. 2A,B). Prior works used Evans Blue Dye (EBD) to assess sarcolemma injury; therefore, we tested for consistency between assays. We observed near complete overlap of myocytes with cytosolic expression of endogenous IgM or IgG proteins and those containing EBD (Fig. S1A,B). We also tested the effects of chronic intermittent isoproterenol (2 mg/kg/day for 10 consecutive days) treatments on myocardial damage (Fig. 1B). Importantly, survival rates were 100% in *mdx* and wild-type mice treated with isoproterenol. Twenty-four hours after the final isoproterenol injection, the area of the dystrophic myocardium occupied by IgM^+^ cardiac myocytes had returned to control levels (Fig. 2A; Fig. S1C). In contrast, myocardial damage was negligible after acute and chronic isoproterenol treatments in wild-type mice (Fig. 2A,B; Fig. S1C). We then co-labeled myocardial sections with anti-mouse serum proteins IgM (900 kDa), IgG (150 kDa) and albumin (65 kDa). Cytosolic expression of all three serum proteins was prominent in the hearts of *mdx* mice following acute isoproterenol treatment (Fig. S1D). No differences in IgG and albumin co-staining were observed. However, not all cells that expressed IgG and albumin also co-expressed IgM, likely due to the passage of small serum proteins through smaller disruptions in the sarcolemma, although the myocardial areas expressing IgG or IgM nearly matched across conditions, indicating that an insignificant number of cells express IgG only (Fig. S1E).

Finally, we measured serum cardiac troponin I (cTnI; also known as TNNI3) concentrations as a separate test of cardiac damage. cTnI is almost exclusively expressed by cardiac myocytes and is a preferred biomarker for evaluation of myocardial injury in DMD (Spurney et al., 2021). Serum cTnI concentrations were greatly increased after acute isoproterenol stimulation in *mdx* relative to wild-type mice (Fig. 2C). In further support of the notion that isoproterenol-induced cardiac myocyte injury is transient in *mdx* mice, serum cTnI levels were not elevated, and were barely detectable, following chronic treatment. Serum cTnI concentrations did not change in wild-type mice in response to isoproterenol stimulation. Consistent with our histological data, these findings suggest that 2 mg/kg/day of isoproterenol is insufficient to induce sarcolemmal damage in wild-type mice. Overall, our data support that dystrophic cardiac myocytes exhibit an acute increased susceptibility to damage, with mechanical stress triggered by isoproterenol challenge.

### Distinct cardiac growth and stress responses to isoproterenol challenge in dystrophin-deficient hearts

**Fig. 3.**
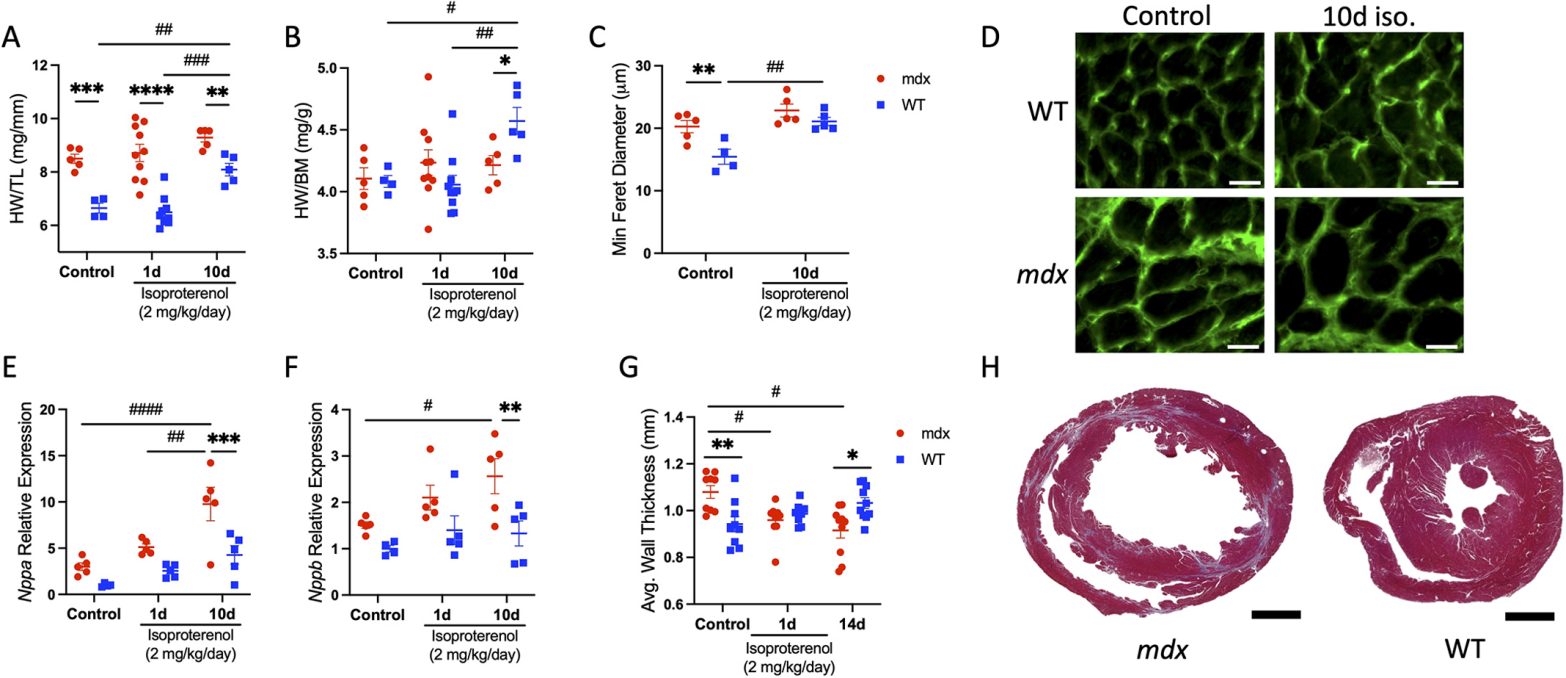
**Distinct cardiac growth and stress responses to β-adrenergic stimulation in dystrophic hearts.** Heart weight normalized to tibia length (HW/TL) and heart weight normalized to body mass (HW/BM) were used as indices of cardiac growth in *mdx* and WT mice treated for 1 (*n*=10 per group) or 10 (*n*=5 per group) days with isoproterenol, and control mice (*n*=5 *mdx* and *n*=4 WT). (A) HW/TL ratio was higher in *mdx* mice than in WT mice in all conditions. Chronic isoproterenol treatment resulted in an increased HW/TL ratio in WT mice only. (B) HW/BM ratio increased with chronic isoproterenol treatment in WT mice relative to that in control and *mdx* mice. (C) Control *mdx* mice have a higher mean minimum ferret diameter than that of WT mice. WT mice treated with isoproterenol for 10 days show significant hypertrophy of cardiac myocytes. (D) Representative images of mid-chamber transverse cardiac sections stained with WGA to visualize cell borders. Scale bars: 20 µm. (E,F) Quantitative PCR (QPCR) analyses of *mdx* and WT ventricles expressed as mRNA levels relative to those of WT control. *Nppa* (E) and *Nppb* (F) mRNA expression increased in *mdx* mice with chronic isoproterenol challenge and relative to that of WT. (G) Ventricular wall thickness was measured at key time points by ultrasound (*n*=10 per genotype). At baseline, wall thickness was greater in *mdx* hearts than in WT hearts. However, in response to isoproterenol treatment, wall thickness decreased in *mdx* mice, becoming less thick than in WT mice at day 14. (H) Mid-chamber coronal sections of *mdx* (left) and WT (right) ventricles stained with Masson's trichrome show characteristic fibrosis and thinning of the free wall in *mdx* mice at day 14. Scale bars: 1 mm. Data are presented as mean±s.e.m. All *P-*values are based on two-way ANOVA with Tukey's multiple comparison test. *P<0.05, **P<0.01, ***P<0.001 and ****P<0.0001 versus WT within a treatment condition. ^#^*P*<0.05, ^##^*P*<0.01, ^###^*P*<0.001 and ^####^*P*<0.0001 between treatment groups within a genotype.

Because previous investigations have shown that isoproterenol stimulation induces cardiac stress and pathological growth (Boluyt et al., 1995), we compared its effects on indices of cardiac growth and stress in wild-type and *mdx* mice. We found that body weight was significantly higher in *mdx* mice (mean±s.e.m., 31.7±0.66 g) than in wild-type mice (26.3±0.61 g), likely due to pseudo-hypertrophy of dystrophic skeletal muscles (Coulton et al., 1988; Lynch et al., 2001). Therefore, we used heart weight normalized to tibia length (Fig. 3A) or body weight (Fig. 3B) as heart weight indices. Heart weight to tibia length ratio was higher in *mdx* mice than in wild-type mice across all treatment conditions (Fig. 3A). In wild-type mice with chronic isoproterenol stimulation, heart weight to tibia length (Fig. 3A) and heart weight to body weight (Fig. 3B) ratios increased 27% and 13% relative to those of control, respectively. Heart weight indices were unaffected by isoproterenol treatment in *mdx* mice. The resulting isoproterenol-stimulated increase in wild-type heart weight to body weight ratio exceeded that in *mdx* mice by 9%. To determine whether the effects on heart mass were due to differences in myocyte hypertrophy, we measured the minimum ferret diameter of cardiac myocytes in wild-type and *mdx* hearts. Indeed, we found that *mdx* (20.27±0.98 μm) cardiac myocytes were 31% larger than wild-type (15.45±1.2 μm) cardiac myocytes under control conditions. However, in response to isoproterenol treatment, hypertrophic growth was only observed in wild-type cardiac myocytes (21.1±0.66 μm; Fig. 3C,D), increasing by 37% relative to control. These data indicate that, under control conditions, increased dystrophic cardiac myocyte size may account for a higher heart weight to tibia length ratio in *mdx* compared to wild-type mice. However, isoproterenol did not induce cardiac myocyte hypertrophy and affect heart weight in *mdx* mice, suggesting that wild-type and dystrophin-deficient cardiac myocytes respond distinctly to β-adrenergic stimulation.

Pathological cardiac growth and remodeling are also marked by increased expression of fetal genes *Nppa* and *Nppb*, encoding atrial natriuretic peptide and brain natriuretic peptide, respectively (Frey and Olson, 2003; Maillet et al., 2013). Quantitative PCR (QPCR) analysis was performed on ventricles from control and isoproterenol-treated wild-type and *mdx* mice. Chronic isoproterenol stimulation increased *Nppa* (Fig. 3E) and *Nppb* (Fig. 3F) mRNA expression by 3.3- and 1.7-fold, respectively, in *mdx* mice compared to control. Consistent with the notion that isoproterenol-induced pathologies are exacerbated in dystrophin-deficient cardiac myocytes, *Nppa* and *Nppb* mRNA levels were 2.3- and 1.9-fold higher, respectively, in *mdx* mice with chronic isoproterenol treatment relative to those in wild-type mice with chronic isoproterenol treatment.

We also observed relative changes in myocardial wall thickness throughout the study. Using a four-dimensional ultrasound (4DUS) analysis technique, we characterized both the endocardial and epicardial surfaces using a three-dimensional dynamic mesh of the left ventricle over a characteristic cardiac cycle as described previously (Damen et al., 2021; Dann et al., 2022). Using these meshes, we calculated an ‘average’ myocardial thickness by finding the mean distance from each point on the endocardial surface and its corresponding point on the epicardial surface at peak systole. We discovered an overall decrease in left-ventricular wall thickness in *mdx* mice from baseline (1.08±0.08) to day 14 (0.92±0.10) following the 10 day course of isoproterenol treatments (*P=*0.029; Fig. 3G). In the wild-type group, we observed a slight, but not significant, increase in average myocardial thickness from baseline (0.94±0.10) to day 14 (1.03±0.07; *P=*0.088; Fig. 3G). This difference is also highlighted in the comparison between *mdx* and wild-type mice in which *mdx* mice had a greater average myocardial thickness at baseline (*P*=0.006) but a reduced thickness at day 14 (*P*=0.010) after isoproterenol challenge (Fig. 3G). Reduced ventricular wall thickness was also apparent qualitatively on histological examination at end stage in *mdx* hearts (Fig. 3H; Fig. S2).

### Isoproterenol promotes fibrotic replacement of the dystrophic myocardium

**Fig. 4.**
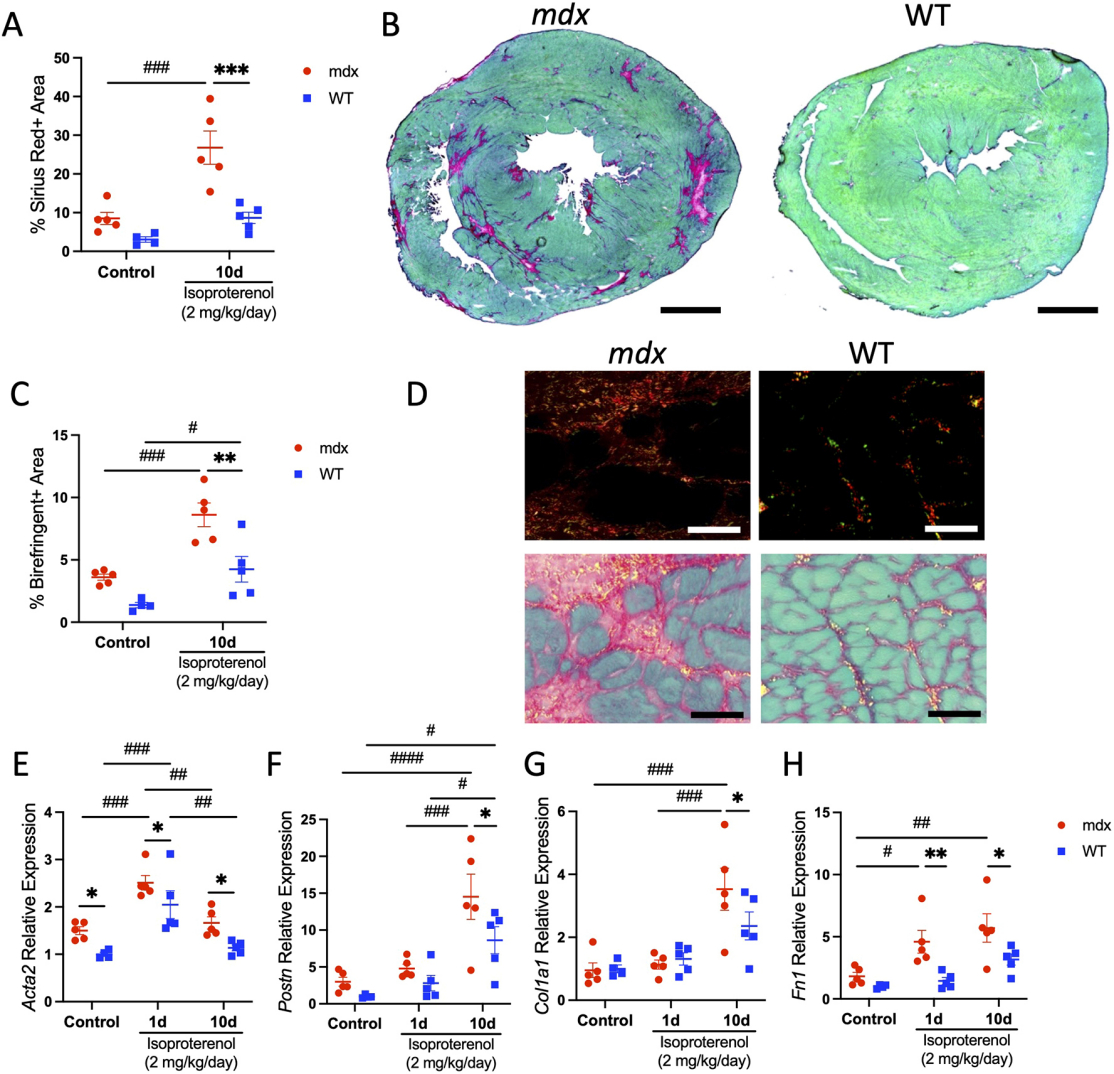
**Isoproterenol promotes fibrotic replacement of the dystrophic myocardium.** (A-G) Fibrotic area was measured on mid-chamber cross-sections stained with Sirius Red Fast Green (SRFG) in control (*n*=5 *mdx* and *n*=4 WT) and chronic isoproterenol-treated (*n*=5 per genotype) mice. (A,B) The area of the myocardium occupied by collagen (red stain) is increased in *mdx* mice (left in B) after chronic isoproterenol challenge relative to that in control and isoproterenol-treated WT mice (right in B). Scale bars: 1 mm. (C,D) Collagen density was visualized by polarized light microscopy in isoproterenol-treated *mdx* and WT mice. (C) Chronic isoproterenol treatment increased areas of birefringence in *mdx* and WT mice. (D) Fibrotic lesions of chronic isoproterenol-treated *mdx* mice contained prominent birefringent areas (red and orange), indicating the presence of densely bundled collagen fibers (top row). Representative overlay images of brightfield and polarized light *mdx* and WT ventricles with birefringent areas pseudo-colored yellow to enhance image contrast in image overlays (bottom row). Scale bars: 50 μm. (E-H) QPCR analyses of transcripts encoding markers of activated fibroblasts (E,F) and connective tissue proteins (G,H). (E) *Acta2* mRNA levels were elevated in *mdx* mice relative to those in WT mice at baseline and with isoproterenol stimulation. (F) Induction of *Postn* transcripts was greater with isoproterenol stimulation in *mdx* mice than in WT mice. (G,H) Genes encoding extracellular matrix components *Col1a1* (G) and *Fn1* (H) were further increased in *mdx* mice relative to those in WT mice with isoproterenol treatment. Data are expressed as mRNA levels relative to those of the WT control group and presented as mean±s.e.m. All *P-*values are based on two-way ANOVA with Tukey's multiple comparison test. *P<0.05, **P<0.01 and ***P<0.001 versus WT within a treatment condition. ^#^*P*<0.05, ^##^*P*<0.01, ^###^*P*<0.001 and ^####^*P*<0.0001 between treatment groups within a genotype.

The increased occurrence of sarcolemmal damage with isoproterenol treatment in *mdx* mice could lead to cardiac myocyte cell death and fibrotic replacement of the myocardial tissue. We tested for changes in fibrosis by histology. The proportion of the myocardium occupied by collagen was increased in *mdx* mice (26.8%±4.30) with chronic isoproterenol treatment compared to that in control *mdx* mice (8.5%±1.57; Fig. 4A,B). Polarized light microscopy of myocardial areas positive for Sirius Red showed large birefringent areas, indicating more densely bundled collagen strands in isoproterenol-treated *mdx* relative to wild-type mice (Fig. 4C,D). Chronic isoproterenol treatment also increased birefringent area in wild-type mice.

Next, we examined the expression of pro-fibrotic transcripts and their dynamics in response to isoproterenol challenge. QPCR analysis was performed to test for changes in transcripts encoding major extracellular matrix remodeling markers and connective tissue proteins. Alpha smooth muscle actin (*Acta2*) is a frequently used marker of cardiac myofibroblasts (Leslie et al., 1991). We observed a 1.6-, 1.2- and 1.5-fold increase in *Acta2* mRNA expression in *mdx* compared to wild-type mice under control, acute and chronic isoproterenol stimulation conditions, respectively (Fig. 4E). *Acta2* mRNA expression levels were highest after acute isoproterenol stimulation, increasing by 1.7- and 2-fold relative to control in *mdx* and wild-type mice, respectively. Periostin (*Postn*) encodes a matricellular protein also produced by activated cardiac fibroblasts, the activity of which is linked to fibrosis in muscular dystrophy (Kanisicak et al., 2016; Lorts et al., 2012). Chronic isoproterenol treatment induced a 4.9- and 8.6-fold increase in *Postn* gene expression in *mdx* and wild-type mice relative to control, respectively (Fig. 4F). However, after chronic isoproterenol stimulation, *Postn* mRNA expression was 1.7-fold higher in the ventricles of *mdx* mice than in those of wild-type mice. Collagen type 1 (*Col1a1*) and fibronectin (*Fn1*) encode extracellular matrix components comprising fibrotic scar. *Col1a1* mRNA increased 3.5-fold in *mdx* mice with chronic isoproterenol treatment relative to control, and the response was 1.5-fold higher than in wild-type mice under identical conditions (Fig. 4G). In addition, *Fn1* mRNA expression increased 2.5- and 3.1-fold in *mdx* mice with acute and chronic isoproterenol treatment, respectively, relative to control (Fig. 4H). The relative changes in *Fn1* transcripts were 3.2- and 1.8-fold higher in *mdx* compared to wild-type mice with acute and chronic isoproterenol treatment, respectively.

### Left-ventricular function and cardiac reserve decrease in *mdx* mice in response to isoproterenol challenge

**Fig. 5.**
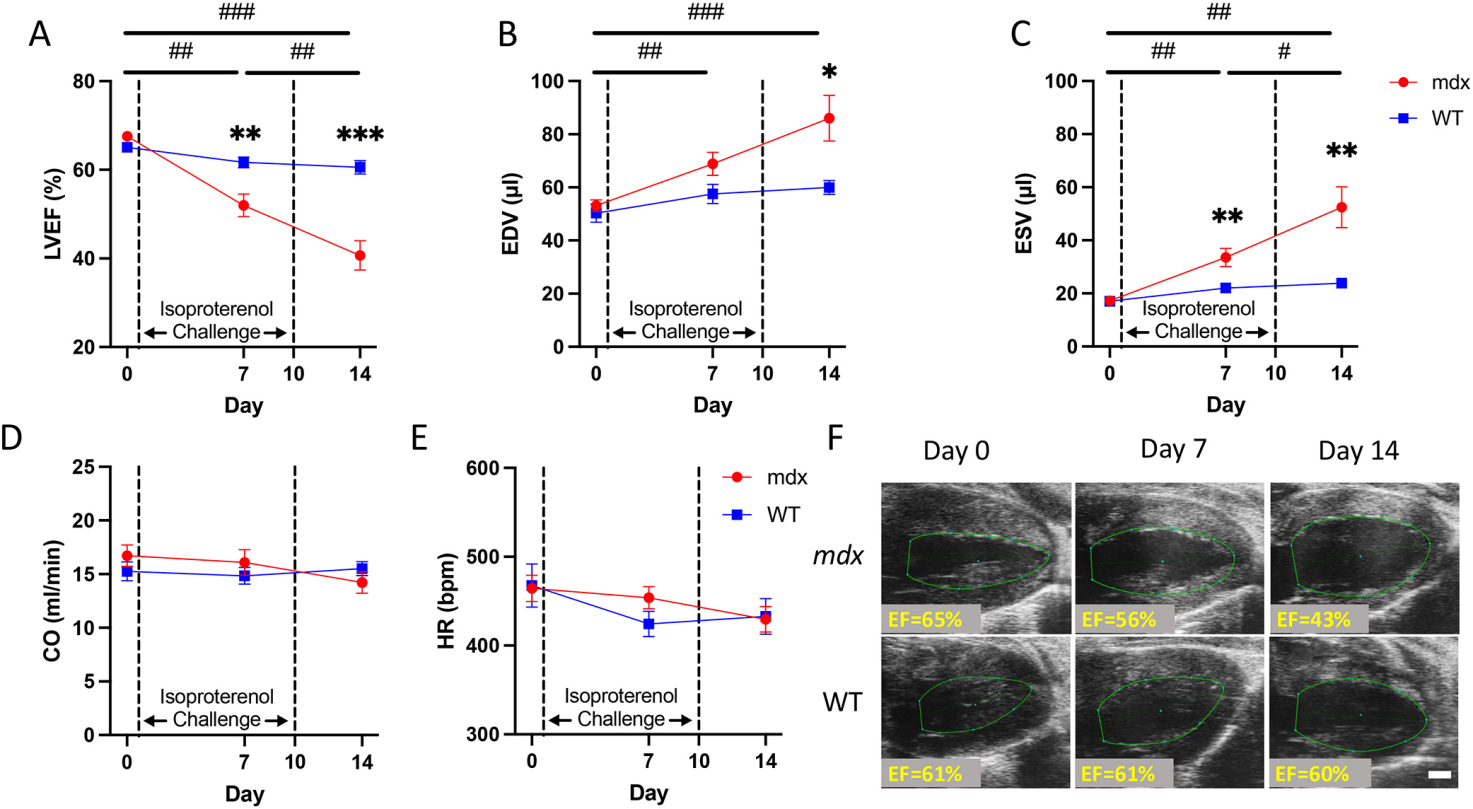
**Cardiac function decreases with isoproterenol injury in dystrophic hearts.** (A) Left-ventricular ejection fraction (LVEF) in *mdx* mice was significantly reduced by day 7 and further reduced at day 14. (B,C) The left-ventricular end-diastolic volume (EDV; B) and end-systolic volume (ESV; C) in *mdx* mice were significantly greater than those in WT mice at day 14, signifying left-ventricular dilation. (D,E) There were no significant changes in the cardiac output (CO; D) or heart rate (HR; E) in either the WT or *mdx* group. (F) 2D ultrasound parasternal long-axis images at peak systole for both *mdx* and WT mice at day 0, day 7 and day 14 with LVEF label (EF). Scale bar: 1 mm. These findings suggest that the *mdx* mice exhibited dilated cardiomyopathy by day 14 when given an isoproterenol challenge. Data are presented as mean±s.e.m. All *P*-values are based on two-way ANOVA with Tukey's multiple comparison test. *P<0.05, **P<0.01 and ***P<0.001 versus WT at a specified timepoint. ^#^*P*<0.05, ^##^*P*<0.01 and ^###^*P*<0.001 *mdx* difference between timepoints. bpm, beats/min.

To determine how isoproterenol injury affected left-ventricular function *in vivo*, we used both 2D and 4D high-frequency ultrasound imaging (Fig. 1C). Using 2D high-frequency ultrasound imaging, we observed a progressive decrease in left-ventricular ejection fraction (LVEF) in *mdx* mice from baseline (68±1%), day 7 (52±3%) and day 14 (41±3%), whereas we noted non-significant changes in wild-type mice during the same period with daily subcutaneous isoproterenol (2 mg/kg/day) administration from day 1 to day 10 (Table 1; Fig. 5A). End-diastolic volume (EDV) also increased in *mdx* mice from 53±2 µl at baseline to 86±9 µl at day 14 (*P*=0.01), as did end-systolic volume (ESV) from 17±1 µl at baseline to 53±8 µl at day 14 (*P*<0.001). There were similarly no significant changes in EDV or ESV for the wild-type group over the course of the study (Fig. 5B,C). Volumetric changes at end systole can also be qualitatively observed on imaging (Fig. 5F, representative images). There were no significant changes in cardiac output (CO) or HR over time for either group (Fig. 5D,E).

**Table 1.**
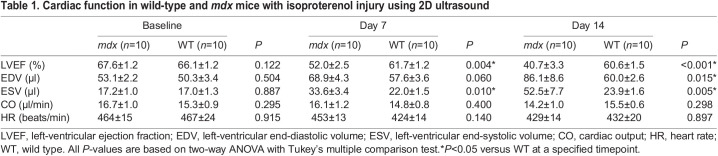
Cardiac function in wild-type and *mdx* mice with isoproterenol injury using 2D ultrasound

**Fig. 6.**
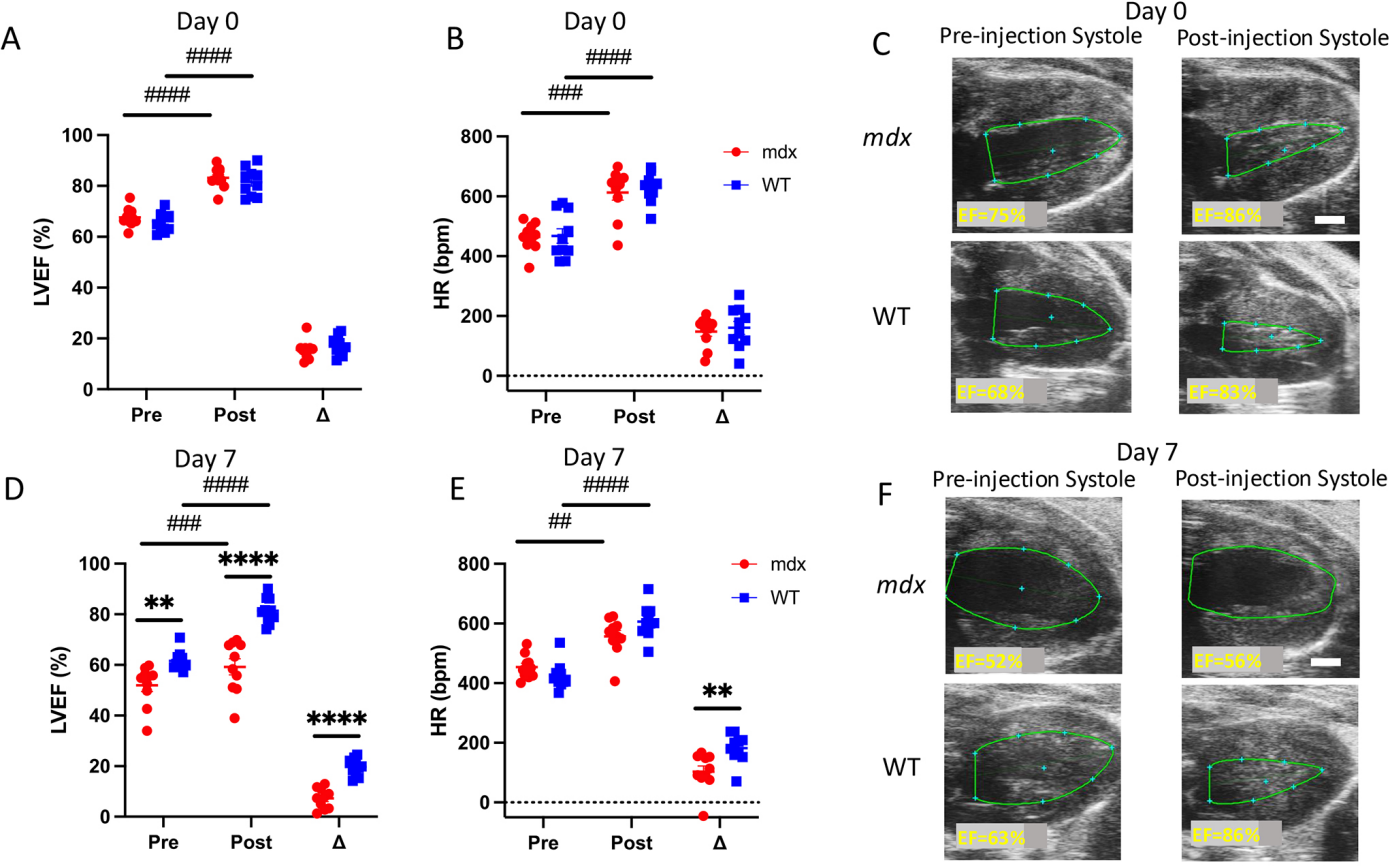
**Cardiac response to isoproterenol challenge decreases over time in dystrophic hearts.** (A,B) The initial compensatory response to an isoproterenol administration measured at exactly 1 min before and 1 min after the injection was apparent in the increase in HR and LVEF in both *mdx* and WT mice. (C) In the 2D ultrasound images of the left ventricle, this compensation can be seen as endocardial walls coming close together during systole. (D,E) After 7 days of exposure to isoproterenol injury, this compensatory response is impaired in the *mdx* mice, in which the ΔLVEF (D) and ΔHR (E) are reduced in comparison to those in WT. (F) There is little to no qualitative change in the left-ventricular chamber in the *mdx* mice compared to WT mice immediately after isoproterenol injection. After prolonged exposure to an isoproterenol challenge, the ability of *mdx* mice to compensate for a single injection is impaired in comparison to the robust compensation of WT mice. Data are presented as mean±s.e.m. All *P-*values are based on two-way ANOVA with Tukey's multiple comparison test. **P<0.01 and ****P<0.0001 versus WT. ^##^*P*<0.01, ^###^*P*<0.001 and ^####^*P*<0.0001 difference between pre- and post-injection.

We also used ultrasound to observe the acute effects of isoproterenol injection for the *mdx* and wild-type mouse groups. At baseline, we observed and measured the cardiac response just prior to isoproterenol administration and ∼1 min following injection. Although we observed a significant increase in absolute LVEF for both groups following isoproterenol injection at baseline (*P*<0.001), we found no differences in ΔLVEF (change in LVEF 1 min following isoproterenol injection) for both *mdx* (15.6±3.7%) and wild-type (15.4±5.6%) mouse groups (*P*=0.95) (Fig. 6A,C). We also observed a robust increase in HR immediately following isoproterenol administration, compared to baseline, for both *mdx* and wild-type mice, with no significant difference in ΔHR between groups (Fig. 6B). However, at day 7, we did see a significant decrease in the ΔLVEF in the *mdx* group (7.3±4.0%) compared to that in the wild-type group (19.7±3.7%; *P*<0.001; Fig. 6D,F; Table S1). We also saw a significant decrease in ΔHR in the *mdx* (*P*=0.01) compared to wild-type group (Fig. 6F). Similarly, when comparing cardiac function metrics at baseline prior to and 1 min following isoproterenol injection, we saw no significant differences in ΔEDV, ΔESV and ΔCO between the *mdx* and wild-type groups (Table S1). At day 7, there were differences between the *mdx* and wild-type groups for ΔESV (*P*=0.01) and ΔCO (*P*=0.03) (Table S2).

### 4D ultrasound myocardial strain magnitude decreases in dystrophic hearts with isoproterenol injury

**Fig. 7.**
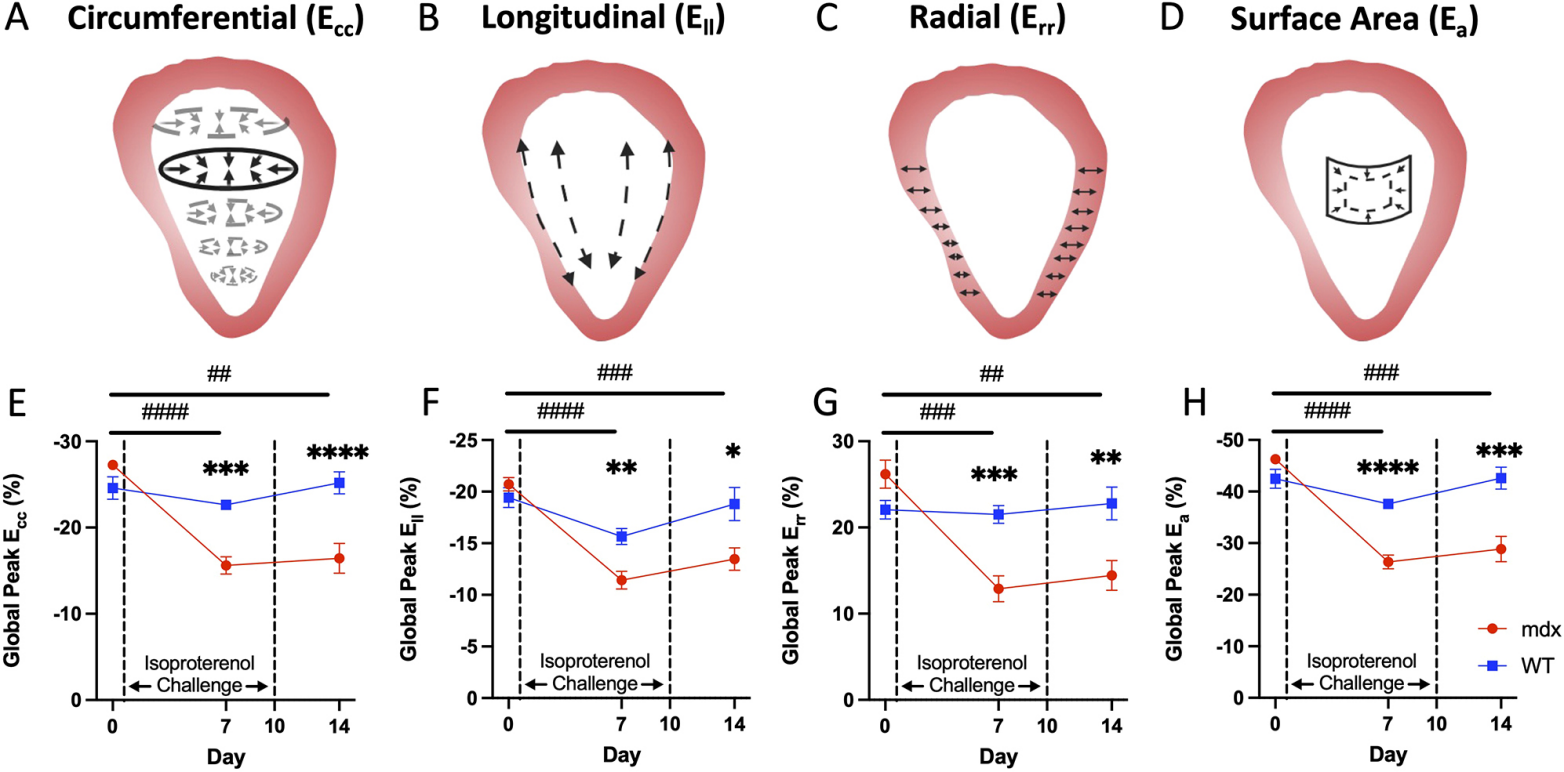
**4D strain magnitude decreases in dystrophic hearts with isoproterenol injury.** (A-D) Global circumferential, longitudinal, radial and surface area strain were calculated with 4D ultrasound (4DUS) measurements. (E-H) In the *mdx* mice with isoproterenol injury, the magnitude of strain was significantly lowered by day 7. There was a slight recovery in ventricular strain by day 14, yet the strain magnitude remains significantly reduced. 4D strain decreases in dystrophic hearts when given an isoproterenol challenge. After the completion of the isoproterenol challenge, there is some functional recovery, but irreversible damage remains. Data are presented as mean±s.e.m. All *P-*values are based on two-way ANOVA with Tukey's multiple comparison test. *P<0.05, **P<0.01, ***P<0.001 and ****P<0.0001 versus WT at a specified timepoint. ^##^*P*<0.01, ^###^*P*<0.001 and ^####^*P*<0.0001 *mdx* difference between timepoints. E_cc_, circumferential strain; E_ll_, longitudinal strain; E_rr_, radial strain; E_a_, surface area strain.

Using our 4D strain technique, we estimated global circumferential (E_cc_), longitudinal (E_ll_), surface area (E_a_) and radial (E_rr_) strain components for *mdx* (*n*=10) and wild-type (*n*=10) mice at baseline, day 7 and day 14 after isoproterenol challenge on days 1-10 (Fig. 7). For example, global average E_cc_ decreased from −27.3±1.0% at baseline to −15.6±3.1 (*P*<0.001) at day 7 and −16.4±5.4 (*P*=0.001) at day 14 in the *mdx* group, but showed no significant changes in the wild-type group [from −24.6±4.1% at baseline to −22.6±1.3% (*P*=0.31) at day 7 and −25.2±4.0% (*P*=0.92) at day 14 (Fig. 7A)]. Global E_cc_ magnitude was also significantly decreased in *mdx* mice compared to wild-type mice at day 7 (*P*<0.001*)* and day 14 (*P*<0.001). We noticed a similar pattern of strain differences between *mdx* and wild-type groups for global E_ll_, E_a_ and E_rr_ (Fig. 7B-D), as well as for regional strain patterns (Table S3).

## DISCUSSION

Our study results demonstrate that mechanical stress induced by β-adrenergic stimulation advances disease state in *mdx* mice, recapitulating cardiac pathologies present in DMD. Our findings show that, before the acute onset of cardiac disease in *mdx* mice, cardiac myocytes are transiently susceptible to isoproterenol-induced injury, which is associated with increased fibrotic replacement of the myocardial tissue. The low-intensity isoproterenol stress protocol used here was insufficient to induce serum or histological markers of cardiac injury in normal hearts, emphasizing the high sensitivity of dystrophin-deficient cardiac myocytes to mechanical stress. Additionally, the induction of transcripts encoding markers of cardiac stress, remodeling and connective tissue proteins were potentiated in response to isoproterenol stimulation in dystrophic hearts. Distinctly, isoproterenol promoted thinning of the left-ventricular wall and increased systolic and diastolic chamber volumes in *mdx* mice, whereas cardiac hypertrophy occurred in wild-type mice. LVEF progressively declined, and cardiac reserve capacity and magnitude of myocardial strain both also decreased, in *mdx* mice in response to isoproterenol exposure. Finally, the isoproterenol challenge did not affect mortality, and therefore dynamic and longitudinal changes in cardiac morphology and function are without survival bias. Collectively, these data support a reproducible model in which pharmacologically induced mechanical stress causes acute myocyte injury and progressive left-ventricular dysfunction evolving into a form of dilated cardiomyopathy.

Relationships between isoproterenol-induced mechanical stress and dystrophic myocardial injury have been known for many years (Danialou et al., 2001). The magnitude of injury that we report, over 8% of the myocardium, is consistent with previous studies that also injected isoproterenol (Danialou et al., 2001; Yue et al., 2004). Several investigations have shown that injections of higher concentrations of isoproterenol can result in a more severe injury that is also associated with a higher incidence of mortality in *mdx* mice (Kamdar et al., 2020; Meyers et al., 2020; Strakova et al., 2014). Damaged myocytes are cleared from the dystrophic heart within 1 week of a single isoproterenol injection (Meyers et al., 2019). Our observation that cardiac injury was negligible in *mdx* mice after ten daily isoproterenol treatments supports that its effects on dystrophic myocyte damage are transient. These data suggest poor responsiveness to continuous isoproterenol doses, possibly due to receptor downregulation or impaired signal transduction. In support of this observation, low incidences of cardiac injury were also reported in *mdx* mice implanted with osmotic pumps for 14 or 28 days of continuous isoproterenol infusion (Spurney et al., 2011). However, we also find that the expression of cardiac stress genes, *Nppa* and *Nppb*, is highest in *mdx* hearts after chronic isoproterenol treatment. That observation could suggest that dystrophic hearts are in a state of elevated stress despite only an acute susceptibility to sarcolemmal injury with sustained isoproterenol treatment.

Our findings also indicate gross differences in left-ventricular remodeling and morphology in dystrophin-deficient hearts. We observed a higher ratio of heart weight to tibial length in *mdx* relative to wild-type mice. However, this ratio only increased significantly in wild-type mice in response to isoproterenol treatment, whereas it remained relatively unchanged in the *mdx* group. In our 4DUS studies, we observed that global average systolic wall thickness was initially higher in *mdx* mice than that in wild-type mice. Still, isoproterenol treatment led to significant thinning of the ventricular wall in *mdx* mice, whereas there was an insignificant increase in wall thickness in wild-type mice. We also noted decreased left-ventricular wall thickness in *mdx* mice in our histological studies. Furthermore, we found that isoproterenol stimulated an increase in left-ventricular EDV and ESV in *mdx* mice. Owing to reported lethality (Sarma et al., 2010; Spurney et al., 2011), the isoproterenol dose (2 mg/kg/day) used in the present study is considerably lower than standardized doses (30 mg/kg/day) known to induce hypertrophy in normal hearts and likely explains why we observe only a mild hypertrophic phenotype in wild-type mice (Chang et al., 2004; Frey et al., 2004; Keys et al., 2003; Zahabi et al., 2003). Collectively, these data support that the *mdx* model's susceptibility to isoproterenol injury and fibrotic replacement of viable cardiac tissue leads to ventricular dilation.

To our knowledge, this is the first study to use 4D high-frequency ultrasound to evaluate progressive cardiac functional, morphological and kinematic changes caused by mechanical stress-induced injury in dystrophin-deficient hearts *in vivo*. Consistent with previous reports (Au et al., 2011; Li et al., 2014; Quinlan et al., 2004; Spurney et al., 2008; Stuckey et al., 2012), our findings show no significant differences in baseline cardiac function in 10- to 12-week-old *mdx* mice. However, after initiating isoproterenol treatment, LVEF progressively decreased in *mdx* mice. Despite these changes, we found that CO is preserved throughout the study, which may indicate that the functional changes we observe are enough to compensate for metabolic demand. When this demand is challenged acutely with isoproterenol, we begin to see evidence of decompensation, as both ΔHR and ΔCO are significantly decreased in the *mdx* group after 7 days of repeated isoproterenol doses. These results appear consistent with previous work showing that reduced acute cardiac β-adrenergic response is an early indicator of heart disease at the compensatory stage (Li et al., 2014). This reduction in cardiac reserve capacity may occur due to the loss of myocytes with the progressive onset of heart disease or in response to isoproterenol injury in *mdx* mice. We also noted a decrease in 4DUS strain magnitude for global circumferential, longitudinal, radial and surface area strain components in dystrophic hearts in every region examined, globally suggesting that the response to isoproterenol injury affected the entire myocardium. In our histology studies, we observed characteristic diffuse fibrotic lesions throughout the myocardium, potentially accounting for changes in myocardial kinematics measured with strain. Each strain component was reduced at day 7 relative to baseline, but there was no further reduction from day 7 to day 14, as seen with the ejection fraction. One possible explanation for this is that our strain metrics may be able to indicate the full extent of myocardial damage earlier than other global or traditional metrics. Alternatively, this finding could indicate a partial recovery of cardiac contractility at day 14 following the termination of isoproterenol treatments at day 10. Future work will be needed to elucidate these possibilities.

Although others have used isoproterenol to progress cardiac disease state in *mdx* mice (Elsherif et al., 2008; Lu and Hoey, 2000; Sarma et al., 2010; Spurney et al., 2011; Sullivan et al., 2021; Yue et al., 2004), the reported cardiac functional outcomes of isoproterenol injury are highly variable from study to study, in part owing to differences in cardiac readouts, as well as unique isoproterenol protocols differing in dosage, pure versus racemic mixtures, frequency, route of administration and method of delivery affecting comparisons between studies. For example, three sequential dosages of isoproterenol (0.35 mg/kg) over 12 h caused myocardial injury in 10% of the myocardium, and acute systolic and diastolic hemodynamic dysfunction (Yue et al., 2004). However, subsequent studies reporting an identical dose and frequency observed less than 2% injury, suggesting that environmental factors could also contribute to disparities among reports (Meyers et al., 2020). Echocardiographic assessment showed a modest 9% reduction in ejection fraction and a 13% decrease in shortening fraction in *mdx* mice 5 days after completing five daily doses of isoproterenol (3 mg/kg/day) (Sullivan et al., 2021). A decrease in ejection fraction and shortening fraction was seen in *mdx* mice 1 day after implantation of an osmotic pump for continuous delivery of isoproterenol (4 mg/kg/day); however, high mortality rates prevented further characterization (Sarma et al., 2010). Osmotic pump delivery of a high concentration of isoproterenol (30 mg/kg/day) for 7 or 14 days did not affect ejection fraction or shortening fraction in *mdx* mice (Elsherif et al., 2008). In contrast, another report found that *mdx* mice succumbed within 24 h of implantation of pumps delivering 1, 5, 10, 15 and 30 mg/kg/day, but were able to mostly tolerate 0.5 mg/kg/day. Two weeks of delivery at this low dose stimulated an increase in the shortening fraction; however, these changes did not persist, returning to control levels after 4 weeks (Spurney et al., 2011). Thus, systolic dysfunction is not observed in *mdx* mice receiving non-lethal doses of isoproterenol via an osmotic pump, indicating that the mode of delivery may account for some of the variation in functional outcomes between studies. However, the findings in our study are in general agreement with others showing that the injection of isoproterenol promotes systolic dysfunction in *mdx* hearts.

Our study results include several unexpected findings. We were surprised that cardiac injury, IgM inclusion and elevated cTnI concentrations were not prevalent in *mdx* hearts following chronic isoproterenol treatment. Furthermore, we observed a reduction in LVEF of nearly similar magnitude from baseline to day 7 and from day 7 to day 14 in *mdx* mice. Together, those data support that the progressive decline in LVEF is not due to extensive additional cardiac myocyte injury with chronic isoproterenol treatment. Those findings may suggest a link between the accumulation of fibrotic scar in the myocardium and a continued decline in function. Previous work in DMD patients showed that the extent of myocardial fibrosis strongly correlated with LVEF decline (Tandon et al., 2015). Another intriguing possibility that remains to be tested is a feed-forward relationship between isoproterenol-mediated cardiac injury and respiratory stress. Functional impairment of the diaphragm is highly related to pulmonary dysfunction in DMD patients (Pennati et al., 2020). Furthermore, the diaphragm has an important role in modulating hemodynamics and left-ventricular afterload, which could affect the progression of dystrophic heart disease (Salah et al., 2022). Surprisingly, continuous infusion of isoproterenol for 30 days, at doses significantly higher than those used in our study (30 mg/kg versus 2 mg/kg), improved diaphragm function in wild-type mice (Cabrera-Aguilera et al., 2020). However, to our knowledge, a relationship between isoproterenol and dystrophic diaphragm function has not been established. Thus, it is not clear whether isoproterenol affects diaphragm function in *mdx* mice and whether diaphragm dysfunction affects cardiac disease progression.

The *mdx* model of human DMD has been extensively phenotyped, and the limitations of the model, as it relates to cardiomyopathy, have been described. Numerous studies have proposed therapies to ameliorate disease in the *mdx* mouse, even reporting that the mouse has been cured (Nogami et al., 2021; Sullivan et al., 2021; Sun et al., 2020; Wasala et al., 2020). However, these therapies have not been translated to human clinical trials, especially as it relates to cardiomyopathy. Current guidelines suggest a proactive therapeutic approach of therapies largely derived from evidence based on data from large-scale adult cardiomyopathy and heart failure trials (Birnkrant et al., 2018a; Feingold et al., 2017). There is a need for therapies specific to the loss of dystrophin and the subsequent pathophysiologic cascade that may differ from cardiomyopathy of other etiologies (Earl et al., 2023; Sunthankar et al., 2023). Although no murine model can perfectly recapitulate the cardiomyopathy progression in DMD (Spurney et al., 2009; Willmann et al., 2012), this model of β-adrenergic stress-induced injury has benefits that could lead to the rapid discovery and translation of DMD cardiomyopathy-specific therapies to bring to human clinical trials.

### Conclusions

This study reports that our improved method of low-dose β-adrenergic stimulation is pathogenic for the acceleration of cardiac dysfunction in *mdx* mice. The primary, novel finding of this work is that repetitive isoproterenol injections cause a progressive reduction in ejection fraction, reduced acute cardiac β-adrenergic responses and reductions in cardiac strain magnitude, both regionally and globally without causing mortality in our model. Our study also shows that isoproterenol reduced ventricular wall thickness and increased chamber diameter in *mdx* hearts. This is functionally important in the context of DMD in which heart disease progresses to a form of dilated cardiomyopathy. These data highlight the cardiac functional consequences of the acute vulnerability of dystrophin-deficient cardiac myocytes to mechanical stress. An additional strength of this work is the establishment of an experimental platform with a simple intervention, daily subcutaneous isoproterenol injections, and well-defined functional outcomes that could be used to evaluate therapies targeting contraction-induced damage or associated responses in dystrophic hearts.

## MATERIALS AND METHODS

### Mice

All animal experiments were performed according to approved Institutional Animal Care and Use Committee protocols at Indiana University School of Medicine and Purdue University, conforming with federal ethical regulations and AAALAC standards for animal testing and research. C57BL/10ScSn-Dmd^mdx^/J mice (*mdx*) and C57BL/10ScSn/J (wild-type) mice were purchased from The Jackson Laboratory (Bar Harbor, ME, USA) and bred to maintain active colonies or directly used for experimentation.

### Isoproterenol stimulation

Male wild-type and *mdx* mice, aged 10-12 weeks, received subcutaneous injections of isoproterenol (Sigma-Aldrich, I6504) dissolved in saline (2 mg/kg body weight). Mice undergoing acute isoproterenol stimulation received a single injection. Chronic isoproterenol stimulation included ten consecutive daily injections of isoproterenol (Fig. 1A,B). A control group of mice were treated with an equal volume of saline to control for phenotypes associated with stress due to daily handling and injections (Razzoli et al., 2020). Twenty-four hours after the final injection, mice were sacrificed, and hearts were dissected and separated into apical and mid-chamber coronal sections divided using a stainless steel slicing matrix (Zivic). Sample sizes of *n*=4-5 per genotype for control, *n*=10 per genotype for acute isoproterenol and *n*=5 per genotype for chronic isoproterenol were used per experimental group. For the imaging portion of the study, female wild-type (*n*=10) and *mdx* (*n*=10) mice, aged 10-12 weeks, similarly received daily subcutaneous injections of 2 mg/kg/day isoproterenol (MedChemExpress, HY-B0468). Immediately after completing the baseline imaging, and while the mice were still under isoflurane anesthesia, the first dose of isoproterenol was administered to each mouse to assess cardiac function and physiologic response to the stimulus exactly 1 min post-injection. Each mouse in these groups then received daily injections for 10 days; however, the injection on day 7 was also performed during physiologic monitoring and imaging under anesthesia. After completion of the 10 day isoproterenol challenge, the mice were imaged one final time at day 14 of the study and subsequently sacrificed. At this time, we dissected the heart, then weighed and prepared the samples for histological analysis (Fig. 1C). For EBD experiments, mice received an intraperitoneal injection of EBD (100 mg/kg body weight) dissolved in saline 10 h prior to sacrifice. EBD is a low-molecular-mass azo dye with high affinity for albumin used to assess sarcolemmal injury in myocytes (Matsuda et al., 1995).

### Immunofluorescence

Mid-chamber ventricles were embedded in Optimal Cutting Temperature compound (OCT; Thermo Fisher Scientific), frozen in liquid nitrogen-cooled isopentane, and cut to 10 µm thickness. To assess sarcolemmal damage in cardiac myocytes, coronal sections were fixed in ice-cold acetone for 10 min. Sections were rinsed with PBS+0.05% Tween 20 (PBST) and blocked in 2% goat serum. Cardiac sections were then washed in PBST and labeled with FITC-conjugated wheat germ agglutinin (WGA; 1:500; Vector Laboratories, FL-1021-5), goat anti-mouse IgM Alexa Fluor 594 (1:1000; Invitrogen, A21044) or goat anti-mouse IgM Alexa Fluor 488 (1:500; Biotium, 20840) for 1 h. Myocardial sections were fixed in ice-cold acetone and blocked in 5% normal goat serum in PBS with 0.1 M glycine, 1% bovine serum albumin, 0.1% cold fish gelatin, 0.1% Triton X-100, 0.05% Tween 20 and 0.05% sodium azide for 1 h. Sections were incubated with rabbit anti-albumin (1:100; Proteintech, 16475-1-AP) overnight. Next, sections were incubated with Alexa Fluor 647-conjugated goat anti-rabbit (1:250; Invitrogen, 21244) for 1 h. Sections were mounted with Prolong Gold Antifade with DAPI (Invitrogen).

### Histopathology

Fibrosis was assessed in myocardial sections by Sirius Red Fast Green (SRFG) stain, as previously reported with modification (Meyers et al., 2019). Briefly, 10 µm mid-chamber coronal sections were fixed in ice-cold acetone for 3 h. Slides were rehydrated in 70% ethanol and rinsed with tap water. Sections were stained in 0.1% Direct Red 80 (Sigma-Aldrich, 365548) and 0.1% Fast Green (Thermo Fisher Scientific, F88-10) in 1.3% Picric Acid (Sigma-Aldrich, P6744) for 25 min and rinsed with tap water. Slides were sequentially dehydrated in 70% ethanol followed by 100% ethanol. Sections were cleared with Neo-Clear and mounted with NeoMount (Millipore). Fibrosis was confirmed with Masson's Trichrome stains as previously described (Schepers et al., 2023).

### Microscopy and analysis

Imaging was performed in Zeiss ZenBlue 3.3 software, and specimens were captured using a Zeiss AxioObserver 7 inverted epifluorescence microscope with motorized stage, and Zeiss Axiocam 105 color and Axiocam 506 monochromatic cameras. Whole-tissue montages of WGA-IgM and SRFG were captured and stitched together with Zen Blue Autofocus and Tiles/Positions Modules. High-magnification multi-color fluorescence images of WGA-IgM-stained sections were captured as a *Z*-stack of consecutive focal planes, deconvolved, and combined using the Zen Blue Z Stack and Extended Depth of Focus Modules. Collagen birefringence was assessed by transmitted light using a Polarizer D condenser fixed at a 90° angle and Analyzer Module Pol ACR P&C.

All images were deidentified and analyzed in a masked manner. Myocardial injury was quantified on sections stained with WGA and immunolabeled with fluorescence dye-conjugated anti-IgM. The area of injury was determined by thresholding the IgM fluorescence-positive area in ZenBlue 3.3 and expressing as a proportion of the total tissue area. To quantify fibrotic area, SRFG images were analyzed by measuring the Sirius Red-positive pixel area using the color threshold function in ImageJ. Fibrotic area was then calculated as the proportion of Sirius Red-positive area to total tissue area.

Quantitative analysis of Masson's Trichrome stain was also performed in ImageJ using the color segmentation plug-in to calculate the percentage of fibrosis relative to healthy tissue.

### RNA isolation and QPCR

The apex of the heart was homogenized in Trizol, and RNA was extracted, separated with chloroform and purified using RNeasy Plus kit with gDNA eliminator columns (Qiagen). RNA quantity and quality were measured using a microvolume NanoDrop One Spectrophotometer (Thermo Fisher Scientific). RNA was then electrophoresed on agarose gels, and RNA quality was further assessed by 28S and 18S ribosome RNA integrity. RNA was reverse transcribed with Maxima H Minus cDNA Synthesis Master Mix (Thermo Fisher Scientific). Reaction and cDNA sample mixes were transferred to reaction plates by automation (Integra Assist Plus) and ran on a Bio-Rad CFX384 QPCR system. QPCR experiments were designed as performed previously using established guidelines for experimental design, data normalization and data analysis to maximize the rigor of quantifying the relative levels of mRNA (Bustin et al., 2009; Nolan et al., 2006; Welc et al., 2019). The expression of each gene in wild-type control samples was set to 1, and other expression values were then scaled to that value. Primers are listed in Table S1.

### Serum cTnI enzyme-linked immunosorbent assay (ELISA)

Blood was collected from the femoral artery at the time of sacrifice and left to clot at room temperature for 30 min. Serum was separated by centrifugation and stored frozen at −20°C. Serum cTnI was measured using a cTnI ELISA Kit (Life Diagnostics, CTNI-1-HS) according to the manufacturer's instructions.

### Ultrasound imaging

Mice were anesthetized with 2% isoflurane in an induction chamber. Once anesthetized, we transferred each mouse to the supine position and secured it to a heated plate with electrodes (FUJIFILM VisualSonics, Toronto, Canada) to monitor HR and respiratory rate. We delivered isoflurane through a nose cone maintained at 1.5% throughout the imaging procedure. Using a rectal temperature probe, we also maintained body temperature near 37±2°C.

Mice were imaged with the Vevo 3100 ultrasound system for small animals (FUJIFILM VisualSonics) using the MX550D linear array transducer (25-55 MHz). We used depilatory cream to remove abdominal and thoracic hair. We obtained transthoracic echocardiographic images of the left ventricle. The 2D images taken included a long- and short-axis ECG-gated kilohertz visualization (LAX EKV and SAX EKV, respectively), as well as long- and short-axis motion mode (LAX m-mode and SAX m-mode, respectively). A 4D image of the left ventricle was taken using a linear step motor by acquiring multiple SAX EKV images with a step size of 0.127 mm between images and a frame rate of 300 Hz. The lower bound of the image extended past the apical epicardium, and the upper bound was set to the ascending aorta.

On day 1 and day 7 of the isoproterenol injection protocol, we performed the full imaging protocol, after which we administered the daily dose of subcutaneous isoproterenol (2 mg/kg) while the mouse was still under anesthesia. Approximately 1 min following the injection, we obtained an LAX EKV image to compare left-ventricular function changes prior to and immediately following isoproterenol challenge.

### Statistical analysis

All data are presented as mean±s.e.m. Statistical significance was calculated by unpaired two-tailed Student's *t*-test, or two-way ANOVA with Tukey's multiple comparison test to determine differences among multiple groups. Differences with *P*<0.05 were considered statistically significant.

### Declaration of generative AI and AI-assisted technologies in the writing process

During the preparation of this work, the authors used ChatGPT (OpenAI, San Francisco, CA, USA) in some minor instances to rephrase and/or summarize text in order to improve readability. After using this tool, the authors reviewed and edited the content as needed and take full responsibility for the content of the publication.

## Supplementary Material

10.1242/dmm.050852_sup1 Supplementary information
