# Supplementary material for: Functional cardiac consequences of β-adrenergic stress-induced injury in a model of Duchenne muscular dystrophy
Source: Dis Model Mech. 2024 Oct 9;17(10):dmm050852. doi: 10.1242/dmm.050852 (PMC11488649; doi:10.1242/dmm.050852)
Supplement: Supplementary information [file dmm-17-050852-s1.pdf]

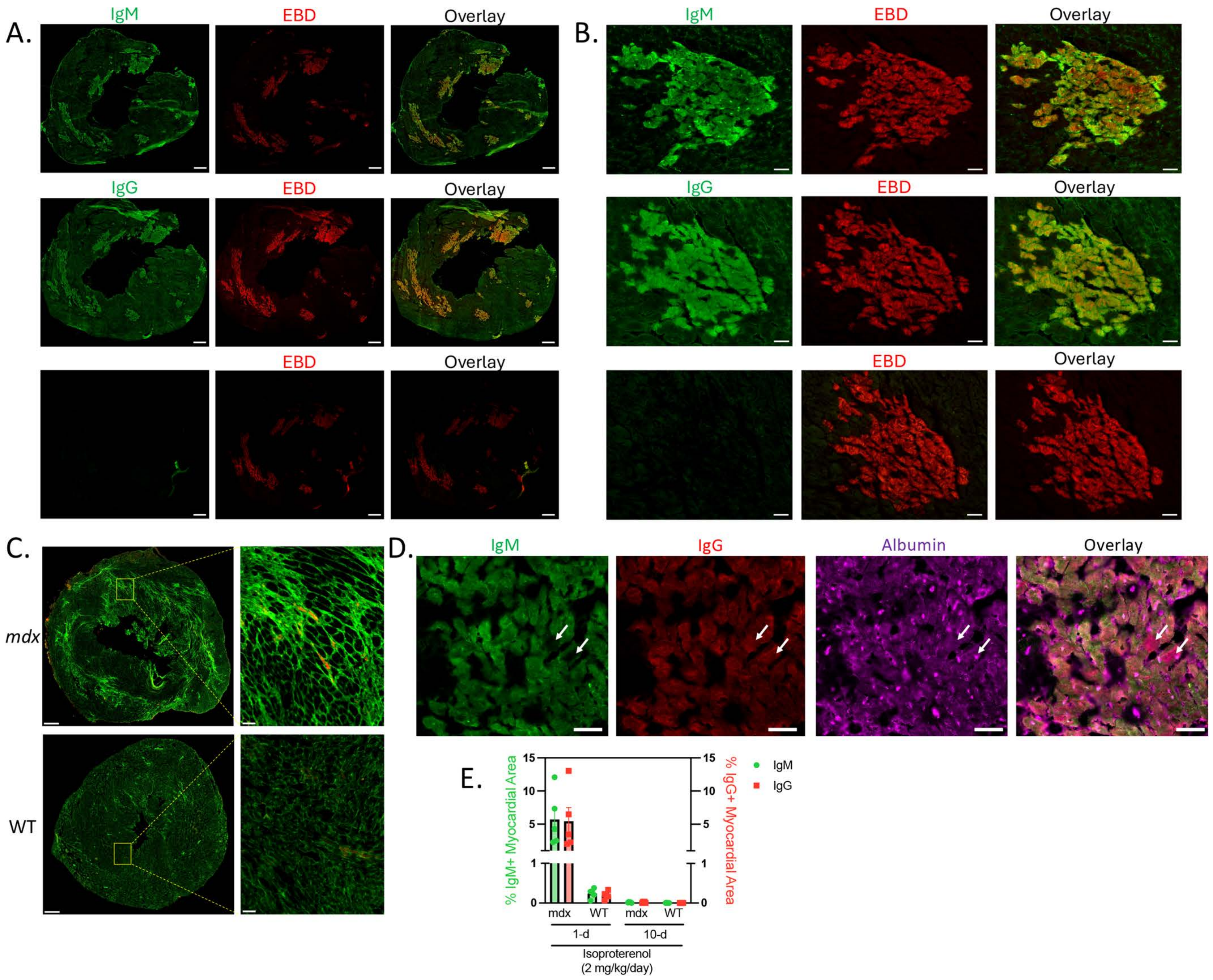

Fig. S1. Cardiac injury in response to isoproterenol stimulation. (A-B) Myocardial sections from *mdx* mice after 1-day of isoproterenol treatment and injected with Evans Blue Dye (EBD) were co-labeled with anti-IgM (top row) or anti-IgG (middle row). (A) Whole tissue montage. Bars=500 µm. (B) High magnification region of interest. Bars=50 µm. (C) Cardiac injury was not sustained with chronic isoproterenol treatment. Representative images of *mdx* (top) and WT (bottom) myocardial cross-sections with chronic isoproterenol treatment immunolabeled with anti-IgM (red) and counterstained with WGA (green). Whole ventricle montages show trace IgM signal in *mdx* and WT ventricles. Bar=500 µm. High magnification images show rare IgM positive signal in *mdx* and WT ventricles. Bars=20 µm. (D) Myocardial sections from *mdx* mice after 1-day of isoproterenol treatment co-labeled with anti-IgM (green), anti-IgG (red), anti-albumin (magenta) or multi-colored image (right). Arrows indicate myocytes immunolabeled with IgG and albumin, but not IgM. Bars=20 µm. (E) Myocardial area occupied by IgM (green) and IgG (red).

Disease Models & Mechanisms • Supplementary information

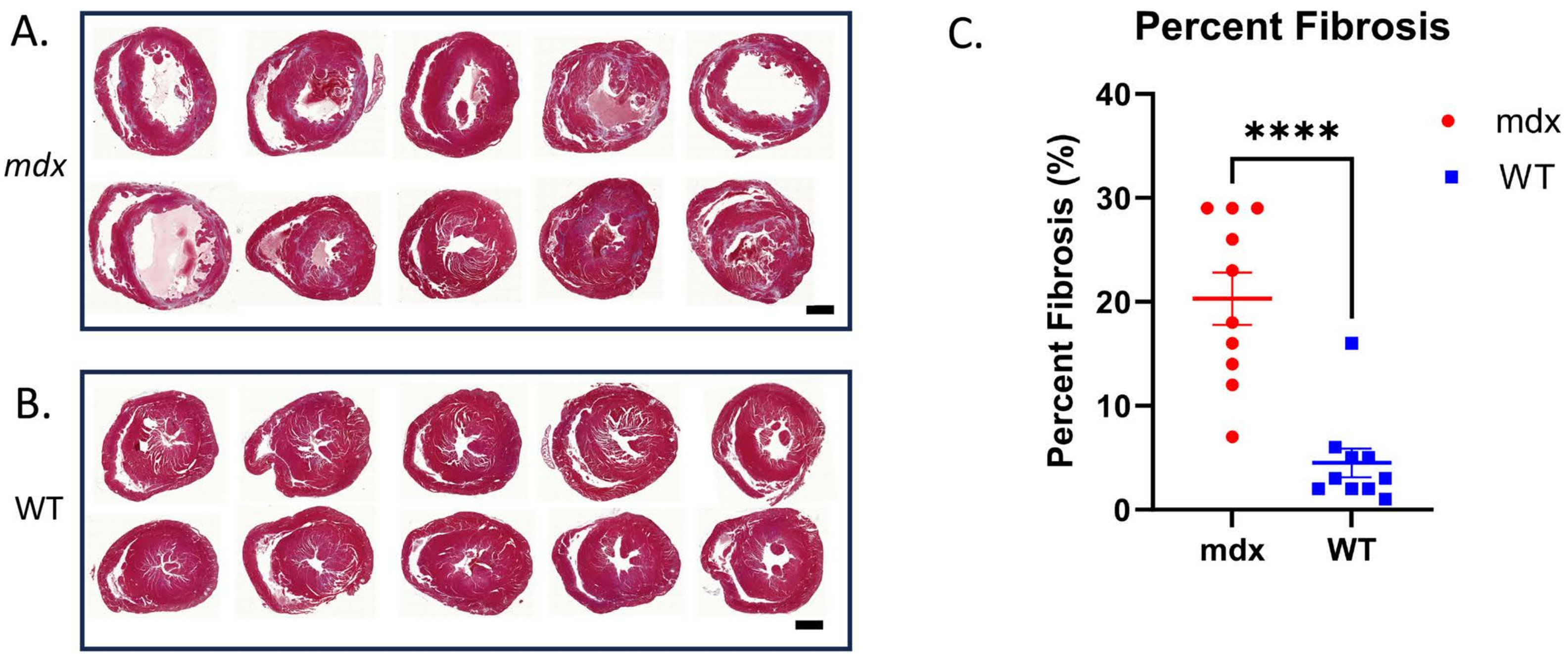

Fig. S2. Fibrosis burden increased in *mdx* mice compared to wild-type with chronic isoproterenol treatment. (A) Masson's trichrome stained mid-ventricle cross sections from *mdx* mice and (B) wild-type mice exposed to chronic isoproterenol treatment. (C) Percent fibrosis differences between groups. *p<0.05, Bar=1 mm.

Table S1. qPCR Primers

Gene	Forward Primer (5'-3')	Reverse Primer (5'-3')
Acta2	CCTGACGCTGAAGTATCCGA	CCTTAGGGTTCAGTGGTGCC
Col1a1	TGTGTGCGATGACGTGCAAT	GGGTCCCTCGACTCCTACA
Fn1	GCTCAGCAAATCGTGCAGC	CTAGGTAGGTCCGTTCCCACTG
Nppa	GCTTCTTCCTCGTCTTGGC	GGGGCATGACCTCATCTTC
Nppb	CTTTATCTGTCACCGCTGGGAG	TGCTGCCTTGAGACCGAAG
Postn	GCTATCTGCGGGAAGAAAACC	GGTCAATAGGCATCACTGCG
Rplp0	GGACCCGAGAAGACCTCCTT	GCTGCCGTTGTCAAACACC
Rsp13	GAGGAACAGAAAGGATAAGGATGC	GGCTGTGGATGACTCATATTTCC

Table S2. Isoproterenol challenge response *inmdx* and wild-type mice with chronic isoproterenol treatment using 2D ultrasound

	Baseline			Day 7		
	mdx (n=10)	WT (n=10)	*p*	*mdx* (n=10)	WT (n=10)	*p*
ΔLVEF (%)	15.6±1.2	15.4±1.8	0.944	7.3±1.3	19.7±1.1	<0.001*
ΔEDV (μL)	-20.5±2.7	-16.5±4.0	0.376	-11.1±2.9	-16.5±2.8	0.230
ΔESV (μL)	-11.6±1.0	-10.6±1.6	0.611	-8.6±1.3	-14.4±1.4	0.005*
ΔCO (μL/min)	-0.2±1.0	1.9±0.9	0.118	2.1±1.0	5.2±1.0	0.023*
ΔHR (beats/min)	148±16	161±22	0.641	103±20	182±15	0.004*

ΔLVEF, left ventricular ejection fraction difference between pre- and post-isoproterenol injection; left ventricular end-diastolic volume difference; ESV, left ventricular end- systolic volume difference; CO, cardiac output difference; HR, heart rate difference.

Table S3. Regional 4DUS strain comparison between wild-type and *mdx* mice

		Baseline			Day 7			Day 14		
		mdx (n=10)	WT (n=10)	*p*	*mdx* (n=10)	WT (n=10)	*p*	*mdx* (n=10)	WT (n=10)	*p*
**Circumferential (E_{cc})**	Basal	-25.1±0.8	-22.8±1.5	0.223	-14.4±1.2	-20.9±0.9	<0.001*	-16.4±1.6	-24.0±1.4	0.003*
	Mid	-27.4±0.8	-25.0±1.1	0.115	-15.4±1.1	-23.6±0.4	<0.001*	-15.7±1.9	-26.2±1.1	<0.001*
	Apical	-27.0±1.1	-25.9±1.3	0.525	-17.1±0.8	-23.4±0.8	<0.001*	-17.2±1.5	-25.3±1.4	0.002*
	Global	-26.5±0.7	-24.6±1.2	0.213	-15.6±0.9	-22.6±0.4	<0.001*	-16.4±1.6	-25.2±1.2	<0.001*
**Longitudinal (E_{ll})**	AFW	-20.2±0.8	-19.5±0.8	0.590	-12.2±0.9	-16.4±0.6	0.002*	-14.0±1.1	-19.9±1.6	0.011*
	A	-19.0±0.5	-18.4±0.9	0.534	-10.4±0.8	-14.7±0.9	0.002*	-12.4±1.2	-17.6±1.6	0.022*
	AS	-18.8±0.6	-17.9±0.9	0.414	-10.2±0.8	-14.3±0.8	0.003*	-12.8±1.0	-16.5±1.5	0.074
	PS	-19.3±0.7	-18.8±1.2	0.727	-10.3±0.7	-13.9±0.8	0.004*	-12.6±1.0	-16.9±1.3	0.028*
	P	-21.3±0.9	-20.8±1.0	0.756	-12.1±0.8	-16.9±0.8	<0.001*	-14.1±1.1	-20.8±1.5	0.004*
	PFW	-21.2±0.9	-21.2±0.9	0.975	-13.4±1.0	-17.7±0.8	0.006*	-14.9±1.0	-21.3±1.5	0.005*
	Global	-20.0±0.7	-19.4±0.9	0.080	-11.4±0.8	-15.7±0.7	0.002*	-13.5±1.0	-18.8±1.5	0.013*
**Radial (E_{rr})**	Basal	14.9±1.4	11.1±0.5	0.030*	7.3±0.7	9.6±0.7	0.034*	7.5±1.1	10.7±1.1	0.076
	Mid	32.8±2.2	27.2±1.5	0.064	15.8±2.0	27.8±1.4	<0.001*	16.9±2.8	28.8±2.3	0.006*
	Apical	32.7±2.0	30.7±2.4	0.562	17.0±2.2	30.0±1.9	<0.001*	21.1±1.9	32.1±3.2	0.014*
	Global	26.0±1.6	21.7±1.0	0.053	12.7±1.4	21.2±1.0	<0.001*	14.2±1.7	22.5±1.8	0.005*
**Surface Area (E_a)**	Basal	-46.7±1.5	-44.9±1.9	0.472	-28.5±1.1	-39.9±1.4	<0.001*	-30.5±2.2	-43.8±2.0	<0.001*
	Mid	-44.7±1.3	-41.5±1.6	0.167	-25.3±1.3	-37.0±0.9	<0.001*	-27.6±2.5	-42.3±2.0	<0.001*
	Apical	-43.3±1.3	-40.3±1.8	0.219	-24.7±1.5	-35.0±1.0	<0.001*	-28.3±2.3	-41.2±2.3	0.002*
	Global	-45.2±1.2	-42.5±1.7	0.252	-26.4±1.2	-37.7±0.9	<0.001*	-28.9±2.3	-42.6±2.0	<0.001*

AFW, anterior free-wall; A, anterior; AS, anterior septal; PS, posterior septal; P, posterior; PFW, posterior free-wall
